# Phytase Production by *Aspergillus niger* CFR 335 and *Aspergillus ficuum* SGA 01 through Submerged and Solid-State Fermentation

**DOI:** 10.1155/2014/392615

**Published:** 2014-01-29

**Authors:** Gunashree B. Shivanna, Govindarajulu Venkateswaran

**Affiliations:** Department of Food Microbiology, Central Food Technological Research Institute, Mysore, Karnataka-570 020, India

## Abstract

Fermentation is one of the industrially important processes for the development of microbial metabolites that has immense applications in various fields. This has prompted to employ fermentation as a major technique in the production of phytase from microbial source. In this study, a comparison was made between submerged (SmF) and solid-state fermentations (SSF) for the production of phytase from *Aspergillus niger* CFR 335 and *Aspergillus ficuum* SGA 01. It was found that both the fungi were capable of producing maximum phytase on 5th day of incubation in both submerged and solid-state fermentation media. *Aspergillus niger* CFR 335 and *A. ficuum* produced a maximum of 60.6 U/gds and 38 U/gds of the enzyme, respectively, in wheat bran solid substrate medium. Enhancement in the enzyme level (76 and 50.7 U/gds) was found when grown in a combined solid substrate medium comprising wheat bran, rice bran, and groundnut cake in the ratio of 2 : 1 : 1. A maximum of 9.6 and 8.2 U/mL of enzyme activity was observed in SmF by *A. niger* CFR 335 and *A.ficuum*, respectively, when grown in potato dextrose broth.

## 1. Introduction

Phytases (EC 3.1.3.8) and phosphatases have a big share in the market due to their widespread application as a feed supplement [1, 2]. Most cereals and legumes are rich in protein and fat but they have antinutritional factors like phytic acid (*myo*-inositol hexakisphosphate) which discourage their use in food. Phytic acid chelates various metals and proteins, thereby diminishing the bioavailability of proteins and nutritionally important minerals such as Ca^2+^, Mg^2+^, and P, Zn^2+^ Fe^2+^ [3]. Phytase (*myo*-inositol hexakisphosphate phosphohydrolase, E.C.3.1.3.8) catalyzes the hydrolysis of phytic acid and its salts (phytates) that generally yield inositol, inositol monophosphate, and inorganic phosphate [4]. The enzymatic degradation of phytic acid will not produce toxic by-products; hence, it is environment friendly [5]. In view of increasing demand for phytase, it is essential to produce phytase in a cost-effective manner using microorganisms.

The production of phytase from fungi has been achieved using three different cultivation methods, namely, solid-state [6], semisolid [7], and submerged fermentation [8, 9]. About 5,000 years ago fungi were cultivated in SSF for the production of food, the oldest known fermentation of rice by *A. oryzae* used to initiate the koji process. Solid-state fermentation (SSF) system has generated a great deal of interest in recent years because it offers several economical and practical advantages including high product concentration, improved product recovery, simple cultivation equipment, and lower plant operational cost [10, 11].

A detailed study on the effect of various cultural conditions for the production of phytase by *Aspergillus niger* CFR 335 in submerged and solid-state fermentation has been carried out [12]. The present study aims at a comparison between the production of phytase enzyme in solid-state and submerged fermentation medium by two fungal strains, *Aspergillus niger *CFR 335 and *Aspergillus ficuum *SGA 01.**

## 2. Experimental Procedures

### 2.1. Strains, Media, and Growth Conditions

*Aspergillus niger *CFR 335 and *Aspergillus ficuum *SGA 01 were cultivated in complete medium (g/L: glucose 10, yeast extract 2.5, malt extract 5, agar 20, pH 5.5 ± 0.2) slants for 3-4 days at 30°C and fully grown slants were stored at 4°C for further use. All the experiments on submerged fermentations were carried out using potato dextrose broth, two different solid substrates, wheat bran, and a combination of wheat bran, rice bran, and groundnut cake (2 : 1 : 1). All the media ingredients were obtained from Hi-Media Chemicals, India. Reagent chemicals were of analytical grade procured from e-Merck, Hi-Media, and Qualigen Chemicals, India, Ltd. and sodium phytate used as a substrate for phytase, standard phytase, and bovine serum albumin were procured from Sigma Chemicals, USA. Fresh wheat bran used in solid-state fermentation was obtained from the Department of Flour Milling Baking & Confectionary Technology, CFTRI, Mysore, India. Rice bran and groundnut cake were procured freshly from a local market.

### 2.2. Studies on the Inoculum Size, Age, and Moisture Level

Effect of different inoculum size (0.25, 0.5, 0.75, 1, 1.5 mL, and 2.0 mL/50 g of solid medium and 100 mL of submerged media) containing 2 × 10^6^ spores/mL on phytase production by* Aspergillus niger *CFR 335 and *Aspergillus ficuum *SGA 01 was studied. In another experiment, the effect of inoculum age on phytase production by* A. niger *CFR 335 and *A. ficuum *SGA 01 in both solid-state and submerged fermentation media was studied using 1–10-day-old inoculum. The effect of moisture level on solid-state fermentation was also studied by varying the moisture from 10 to 80% using sterile distilled water and optimum moisture for maximum phytase production was determined. All the inoculated flasks were incubated at 30°C for 10 days and periodically tested for phytase production.

### 2.3. Studies on Fermentation Temperature, pH, and Time

Effect of fermentation temperature on maximum phytase production was studied by incubating the fungi at temperatures varying from 5 to 60°C at an interval of 5°C. The effect of media pH on maximum phytase production was also studied by setting the media pH between 2.0 and 7.5 at an interval of 0.5. The effect of fermentation time on phytase production was studied by incubating both *A. niger *CFR 335 and *A. ficuum *SGA 01 in submerged (potato dextrose broth) and solid-state (wheat bran) cultivation medium for 10 days and periodically testing the enzyme activity.

### 2.4. Preparation of Solid-State Fermentation (SSF) Medium

In solid-state fermentation studies, two different media including wheat bran (100%) and a mixed medium with combination of wheat bran, rice bran, and groundnut cake (2 : 1 : 1) were used. Solid substrate media were prepared according to the method of Gunashree and Venkateswaran [12]. After cooling to room temperature, sterile solid media were inoculated with 1 mL and 1.5 mL suspensions of *A. niger* CFR 335 and *A. ficuum *SGA 01, respectively, containing 2 × 10^6^ spores/mL. The media were mixed thoroughly using a sterile glass rod and then incubated for 8 days at 30°C in a static inclined position.

### 2.5. Preparation of Submerged Fermentation (SmF) Medium

Aliquots of 100 mL potato dextrose broth (PDB) were taken in 500 mL Erlenmeyer flask and autoclaved for 20 minutes at 121°C and 15 lbs pressure. The media were cooled to room temperature and inoculated with 0.5 and 1 mL suspensions of *A. niger* and *A. ficuum*, respectively, containing 2 × 10^6^ spores/mL. The flasks were incubated for 10 days at 30°C on an orbital shaker at 200 rpm.

### 2.6. Extraction of Crude Enzyme from SSF

Crude enzyme extraction was carried out by the method of Gunashree and Venkateswaran [12] by soaking moldy bran in 1 : 5 w/v 0.2 M acetate buffer at pH 4.5. The flasks were kept in a rotary shaker for 20 minutes at 200 rpm after thorough mixing of the bran with distilled water. The solids were separated from the aqueous solution by filtering through clean muslin cloth. The aqueous solution was centrifuged at 8944 g for 20 minutes at 4°C in a refrigerated centrifuge. The aqueous supernatant was collected and used for further investigation.

### 2.7. Extraction of Crude Enzyme in SmF

Extracellular crude phytase from submerged media was extracted by the method of Gunashree and Venkateswaran [12], which entailed initial filtration through Whatman no. 1 filter paper and centrifugation at 8944 g for 10–15 minutes. The culture broth containing phytase was stored at 4°C and used as crude enzyme preparation for further studies.

### 2.8. Enzyme Assay

Crude enzyme extracted from both solid-state and submerged fermentation media was quantitatively assayed for phytase enzyme as described by the method of Heinonen and Lahti [13]. A standard graph was plotted using potassium dihydrogen phosphate with working concentration ranging from 30 to 360 *μ*M. Protein quantifications were made by the method of Bradford [14] and compared with the standard prepared using bovine serum albumin.

### 2.9. Statistical Analysis

Data are presented as standard error means (±SEM). Comparisons between solid substrate and submerged fermentation between *Aspergillus niger* CFR 335 and *Aspergillus ficuum* SGA 01 were made with analysis of variance [15]. *P* values were considered significant at *P* < 0.05. All statistical tests were carried out using demo version of Graph Pad Prism software.

## 3. Results and Discussion

### 3.1. Inoculum Age

**Figure 1:**
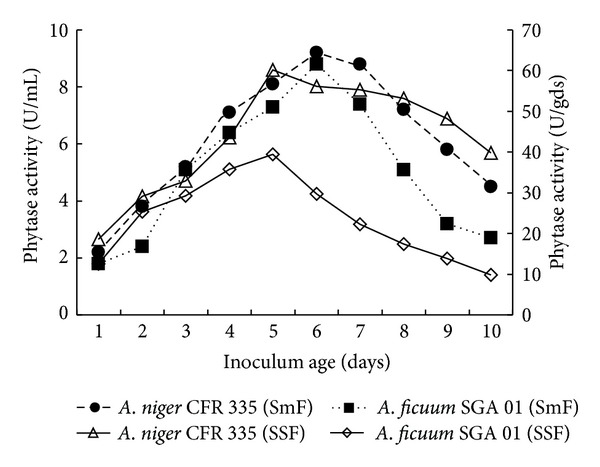
Effect of inoculum age on phytase production by *Aspergillus niger* CFR 335 and *Aspergillus ficuum *SGA 01 in submerged and solid-state fermentation.

Effect of inoculum age, size, and moisture level for maximum phytase production by *Aspergillus niger* CFR 335 and *Aspergillus ficuum *SGA 01 through solid-state and submerged fermentation was studied. Age of inoculum used in fermentation media has an impact on growth of the organism as well as the amount of metabolite that is produced [6]. Studies on the effect of inoculum age showed that the enzyme production rate increased gradually with increase in inoculum age up to six days and declined when older inocula were used. It was found that six-day old culture resulted in maximum phytase production by both *A. niger* CFR 335 and *A. ficuum *SGA 01 under both fermentations (Figure 1). The enzyme activity was 2.2 and 1.8 U/mL when one-day inocula of *A. niger* CFR 335 and *A. ficuum *SGA 01 were used for submerged fermentation with a gradual increase up to 9.2 and 8.8 U/mL with six-day old inocula. In solid-state fermentation, phytase activity was 12.6 and 18.6 U/gds with one-day old inocula and increased up to 60.2 and 39.4 U/gds with six-day old inocula of *A. niger* CFR 335 and *A. ficuum *SGA 01, respectively. About 51 and 69% reductions in phytase activity of *A. niger* CFR 335 and *A. ficuum *SGA 01 in submerged fermentation were observed and a decline of 34 and 75% was observed in solid-state fermentation when ten-day old inocula were used. This decline in enzyme activity with older inocula may be due to reduced metabolic rate. Studies have been extensively carried out on the effect of culture conditions, particularly inoculum age, media composition, and duration of SSF on phytase production by *A. niger *[16]. Ebune et al. [6] have shown 2- and 5-day-old homogenized pellet as inocula for producing the least and the highest amount of enzyme, respectively, whereas in the present investigation, spore suspension was used.

### 3.2. Inoculum Size

**Figure 2:**
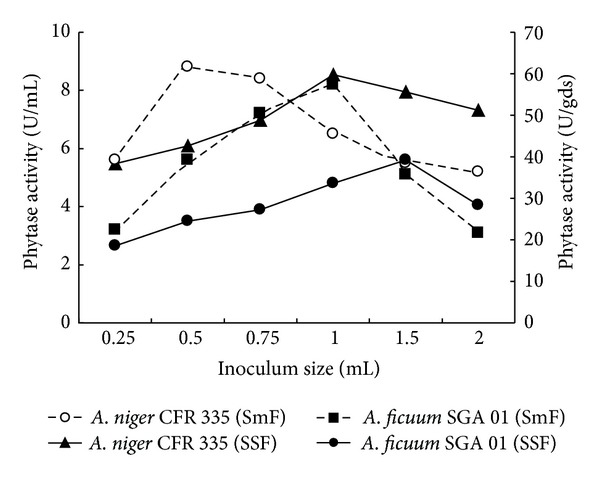
Effect of inoculum size on phytase production by* Aspergillus niger* CFR 335 and *Aspergillus ficuum *SGA 01 in submerged and solid-state fermentation.

Inoculum size also plays an important role in the extent of growth and metabolite production by fungi. The results on different inoculum size indicated that there was a gradual increase in the enzyme synthesis with a maximum activity of 8.8 and 8.2 U/mL with 0.5 and 1 mL of *A. niger* CFR 335 and *A. ficuum* SGA 01 spore suspensions (2 × 10^6^ spores/mL) used for submerged fermentation, respectively. In solid-state fermentation of *A. niger* CFR 335 and *A. ficuum* SGA 01, 1 and 1.5 mL of spore suspensions were optimum for maximum enzyme activities of 59.8 and 39.2 U/gds, respectively, (Figure 2). Minimum enzyme activities of 5.6 and 3.2 U/mL for submerged fermentation and 38.3 and 18.6 U/gds for solid-state fermentation were observed when 0.25 mL of *A. niger* CFR 335 and *A. ficuum* SGA 01 inocula was used, respectively. There was decline of 41 and 62.2% in the submerged and 14.4 and 27.6% in the solid-state fermentation of* A. niger* CFR 335 and *A. ficuum* SGA 01, respectively, when 2 mL inocula were used. This may be due to higher fungal growth that leads to increased competition for nutrients and their fast exhaustion thereby can be retained enzyme production [6]. A similar study on the influence of inoculum level on phytase production has also been carried out earlier [17].

### 3.3. Studies on Moisture Level

**Figure 3:**
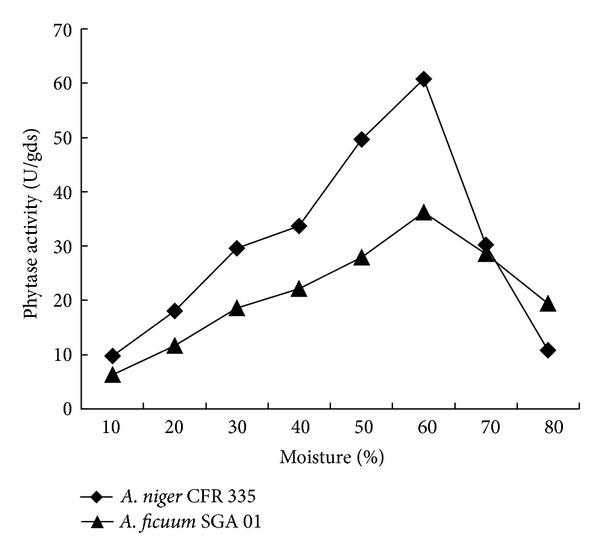
Effect of moisture level on phytase production by* Aspergillus niger* CFR 335 and *Aspergillus ficuum* SGA 01 in solid-state fermentation.

Moisture content is one of the most critical factors for microbial growth and enzyme production in solid-state fermentation [18]. The optimum amount of water varies and must be determined for each microbial system [19]. The present study showed that there was linearity between the enzyme production and moisture levels of solid substrate media up to 60%; further increase in the moisture resulted in reduced enzyme production. The fungi failed to grow in lower moisture levels, while in higher levels, both the fungi grew vegetatively resulting in reduced enzyme yield. (Figure 3). There was 90 and 82% decline in the enzyme activity of *A. niger* CFR 335 and *A. ficuum* SGA 01, respectively, with moisture level beyond 60%. This may be attributed to reduced aeration in the substrate and also due to reduced decomposition rate of total organic matter at the lowest and highest moisture contents [17]. Canola meal has been used for phytase production by *Aspergillus ficuum* with optimum moisture of 64% [6] and by* A. carbonarius *strain with optimum moisture ranging from 53 to 60% [17]. A maximum phytase production was also reported at 60% moisture level [20], which is identical to the present findings.

### 3.4. Effect of Temperature and pH

**Figure 4:**
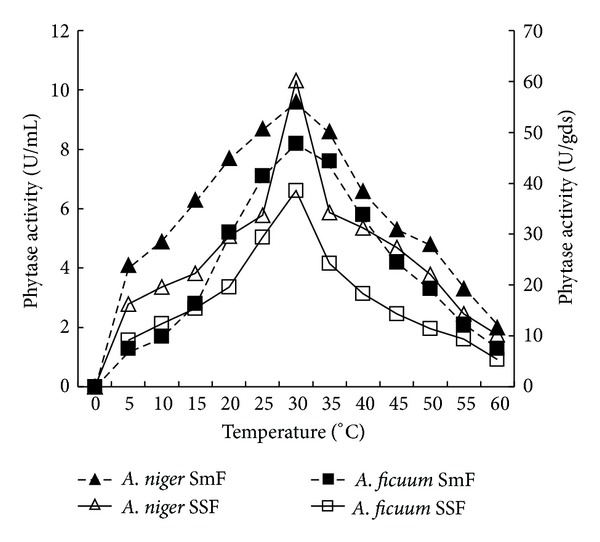
Effect of temperature on phytase production by* Aspergillus niger* CFR 335 and *Aspergillus ficuum* SGA 01 in submerged and solid-state fermentation.

**Figure 5:**
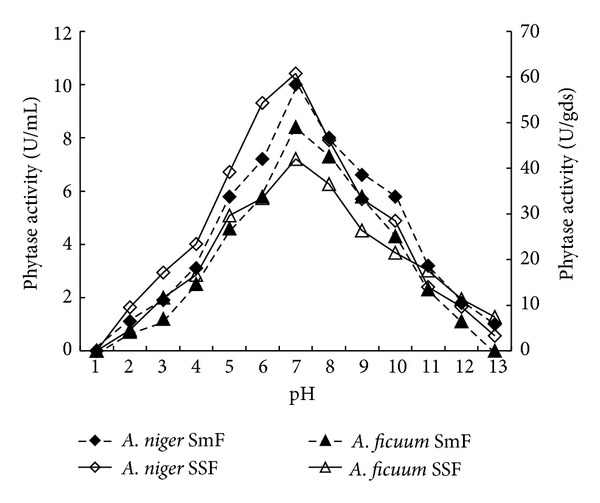
Effect of pH on phytase production by* Aspergillus niger* CFR 335 and *Aspergillus ficuum* SGA 01 in submerged and solid-state fermentation.

Physical parameters like temperature and pH play a vital role in the growth, production, and stability of any microbial metabolite. Results showed linearity between phytase production, and fermentation temperature up to 30°C for both the fungi (Figure 4). There was gradual decline of >80 and 90% in the enzyme production at 60°C under submerged fermentation of *A. niger *CFR 335 and *A. ficuum *SGA 01, respectively. About 70% reduction in enzyme activity was obtained in both the fungi through solid-state fermentation at 60°C. Similarly, there was a linear trend between phytase production and pH up to 4.5 for both the fungi and the enzyme yield was reduced with increase in pH (Figure 5). There was >90% reduction in the enzyme production at pH beyond 4.5. In the present investigation, there was a linear trend between phytase production and pH up to 4.5 for both the fungi and the enzyme yield was reduced with increase in pH. This is in support of earlier reports where they have shown that pH ranging from 4.5 to 6.0 is optimum for filamentous fungi [21–23].

### 3.5. Fermentation Time

**Figure 6:**
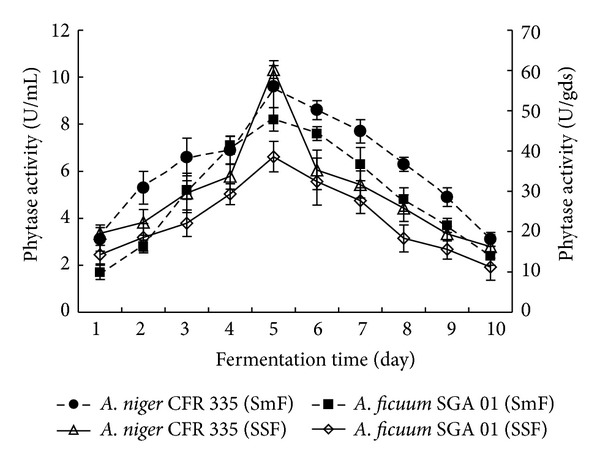
Effect of fermentation time on phytase production by* Aspergillus niger* CFR 335 and *Aspergillus ficuum *SGA 01 in submerged and solid-state fermentation.

The effect of fermentation period on phytase production by *A. niger *CFR 335 and *A. ficuum *SGA 01 in submerged and solid-state fermentation was tested by incubating the fungi for 10 days with periodically testing the enzyme activity. Enzyme activity was found to be increased exponentially with increase in the incubation period. The two fungi grew luxuriantly with abundant conidia in solid substrate medium at an early period of 40–48 h of incubation. Activities of 61 U/gds and 38 U/gds were observed in *A. niger *CFR 335 and *A. ficuum* SGA 01, respectively, on 5th day of solid-state fermentation at 30°C. With subsequent cultivation period, there were 73 and 71% reductions in solid-state fermentation and 62 and 71% reductions, in submerged fermentation of *A. niger *CFR 335 and *A. ficuum* SGA 01 respectively (Figure 6). A similar growth study with respect to phytase production was carried out [23] and found luxuriant growth within 48 h of incubation.

### 3.6. Solid Substrate Media

**Figure 7:**
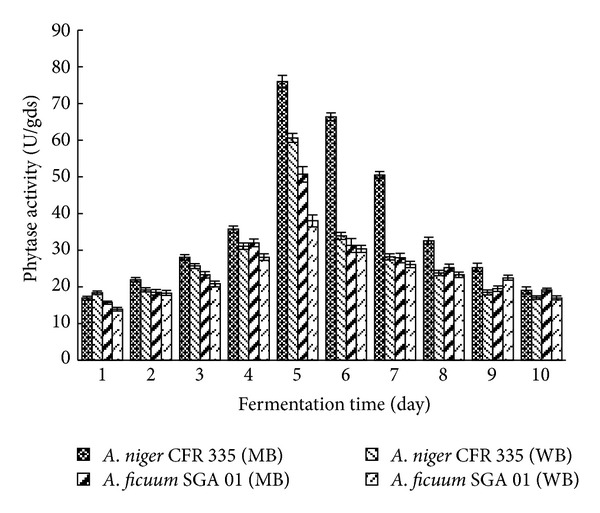
Effect of various solid-substrate media on phytase production by *Aspergillus niger* CFR 335 and *Aspergillus ficuum *SGA 01 in submerged and solid-state fermentation.

The enzyme activities of *A. niger *CFR 335 and *A. ficuum* SGA 01 varied when grown in wheat bran (WB) and mixed solid substrate media (MB) comprising 2 : 1 : 1 proportion of wheat bran, rice bran, and groundnut cake. An activity of 76 U/gds and 51 U/gds was observed in *A. niger *CFR 335 and *A. ficuum* SGA 01, respectively, when grown in mixed solid substrate medium (Figure 7). Enhancement of 24% enzyme activity was observed in mixed medium than in mere wheat bran medium. This increase in the enzyme yield may be due to the presence of nutritionally rich rice bran and groundnut cake. Rice bran contains about 12 to 13% oil and a high level of dietary fibers such as beta-glucan, pectin, and gum. In addition, it also contains 4-hydroxy-3-methoxycinnamic acid (ferulic acid) which may also be a component of the structure of nonlignified cell walls [24]. Groundnut cake is a rich source of protein and monounsaturated fats [25]. A large number of complex high molecular weight polysaccharides such as cellulose, hemicellulose, lignin, and starch are available in wheat bran and serve as additional carbon sources and also increased total phosphorus content prevents phosphate limitation in the culture medium [23]. Similar findings have been shown earlier [26–28].

## 4. Conclusion

It is concluded that *Aspergillus niger *CFR 335 and *Aspergillus ficuum* SGA 01 were capable of accumulating phytase after the onset of sexual growth both in submerged and solid-state fermentation media. However, enzyme accumulation was higher in solid-state fermentation than in submerged fermentation medium, because of freely available aqueous content in submerged medium which only supports the vegetative growth of the fungi. This is in contrast with the natural condition where the microbes grow. Enzyme activity was found to be 3–5 fold higher in SSF than in SmF medium which is higher when compared to earlier reports.

## References

[1] Greiner R, Konietzny U (2006). Phytase for food application. *Food Technology and Biotechnology*.

[2] Vohra A, Satyanarayana T (2003). Phytases: microbial sources, production, purification, and potential biotechnological applications. *Critical Reviews in Biotechnology*.

[3] Ragon M, Hoh F, Aumelas A, Chiche L, Moulin G, Boze H (2009). Structure of *Debaryomyces castellii* CBS 2923 phytase. *Acta Crystallographica Section F*.

[4] Mullaney EJ, Daly CB, Ullah AHJ (2000). Advances in phytase research. *Advances in Applied Microbiology*.

[5] Ciofalo V, Barton N, Kretz K, Baird J, Cook M, Shanahan D (2003). Safety evaluation of a phytase, expressed in *Schizosaccharomyces pombe*, intended for use in animal feed. *Regulatory Toxicology and Pharmacology*.

[6] Ebune A, Al-Asheh S, Duvnjak Z (1995). Production of phytase during solid state fermentation using *Aspergillus ficuum* NRRL 3135 in canola meal. *Bioresource Technology*.

[7] Han YW, Gallagher DJ, Wilfred AG (1987). Phytase production by *Aspergillus ficuum* on semisolid substrate. *Journal of Industrial Microbiology*.

[8] Nair VC, Duvnjak Z (1990). Reduction of phytic acid content in canola meal by *Aspergillus ficuum* in solid state fermentation process. *Applied Microbiology and Biotechnology*.

[9] Ullah AH, Gibson DM (1987). Extracellular phytase (E.C. 3.1.3.8) from *Aspergillus ficuum* NRRL 3135: purification and characterization. *Preparative Biochemistry*.

[10] Becerra M, González Siso MI (1996). Yeast *β*-galactosidase in solid-state fermentations. *Enzyme and Microbial Technology*.

[11] Pandey A, Szakacs G, Soccol CR, Rodriguez-Leon JA, Soccol VT (2001). Production, purification and properties of microbial phytases. *Bioresource Technology*.

[12] Gunashree BS, Venkateswaran G (2008). Effect of different cultural conditions for phytase production by *Aspergillus niger* CFR 335 in submerged and solid-state fermentations. *Journal of Industrial Microbiology and Biotechnology*.

[13] Heinonen JK, Lahti RJ (1981). A new and convenient colorimetric determination of inorganic orthophosphate and its application to the assay of inorganic pyrophosphatase. *Analytical Biochemistry*.

[14] Bradford MM (1976). A rapid and sensitive method for the quantitation of microgram quantities of protein utilizing the principle of protein dye binding. *Analytical Biochemistry*.

[15] Snedecor GW, Cochran WG (1976). *Statistical Methods*.

[16] Krishna C, Nokes SE (2001). Predicting vegetative inoculum performance to maximize phytase production in solid-state fermentation using response surface methodology. *Journal of Industrial Microbiology and Biotechnology*.

[17] Al-Asheh S, Duvnjak Z (1995). The effect of phosphate concentration on phytase production and the reduction of phytic acid content in canola meal by Aspergillus carbonarius during a solid-state fermentation process. *Applied Microbiology and Biotechnology*.

[18] Awad GEA, Elnashar MMM, Danial EN (2011). Optimization of phytase production by *Penicillium funiculosum* NRC467 under solid state fermentation by using full factorial design. *World Applied Sciences Journal*.

[19] El-Batal AI, Abdel Karem H (2001). Phytase production and phytic acid reduction in rapeseed meal by *Aspergillus niger* during solid state fermentation. *Food Research International*.

[20] Vats P, Banerjee UC (2002). Studies on the production of phytase by a newly isolated strain of *Aspergillus niger* var teigham obtained from rotten wood-logs. *Process Biochemistry*.

[21] Casey A, Walsh G (2003). Purification and characterization of extracellular phytase from *Aspergillus niger* ATCC 9142. *Bioresource Technology*.

[22] Oh B-C, Choi W-C, Park S, Kim Y-O, Oh T-K (2004). Biochemical properties and substrate specificities of alkaline and histidine acid phytases. *Applied Microbiology and Biotechnology*.

[23] Papagianni M, Nokes SE, Filer K (1999). Production of phytase by *Aspergillus niger* in submerged and solid-state fermentation. *Process Biochemistry*.

[24] (March 2006). How Nice, Brown Rice: Study Shows Rice Bran Lowers Blood Pressure in Rats. *Science Daily*.

[25] Davies OA, Ezenwa NC (2010). Groundnut cake as an alternative protein source in the diet of *Clarias gariepinus* fry. *International Journal of Science and Nature*.

[26] Ramachandran S, Roopesh K, Nampoothiri KM, Szakacs G, Pandey A (2005). Mixed substrate fermentation for the production of phytase by *Rhizopus* spp. using oilcakes as substrates. *Process Biochemistry*.

[27] Roopesh K, Ramachandran S, Nampoothiri KM, Szakacs G, Pandey A (2006). Comparison of phytase production on wheat bran and oilcakes in solid-state fermentation by *Mucor racemosus*. *Bioresource Technology*.

[28] Bogar B, Szakacs G, Linden JC, Pandey A, Tengerdy RP (2003). Optimization of phytase production by solid substrate fermentation. *Journal of Industrial Microbiology and Biotechnology*.

